# Supplementary material for: Process Optimization for Production of Persimmon Wine with Lower Methanol
Source: Foods. 2024 Feb 28;13(5):748. doi: 10.3390/foods13050748 (PMC10931408; doi:10.3390/foods13050748)
Supplement: Supplementary file 1 [file foods-13-00748-s001.zip › foods-2856413-supplementary.pdf]

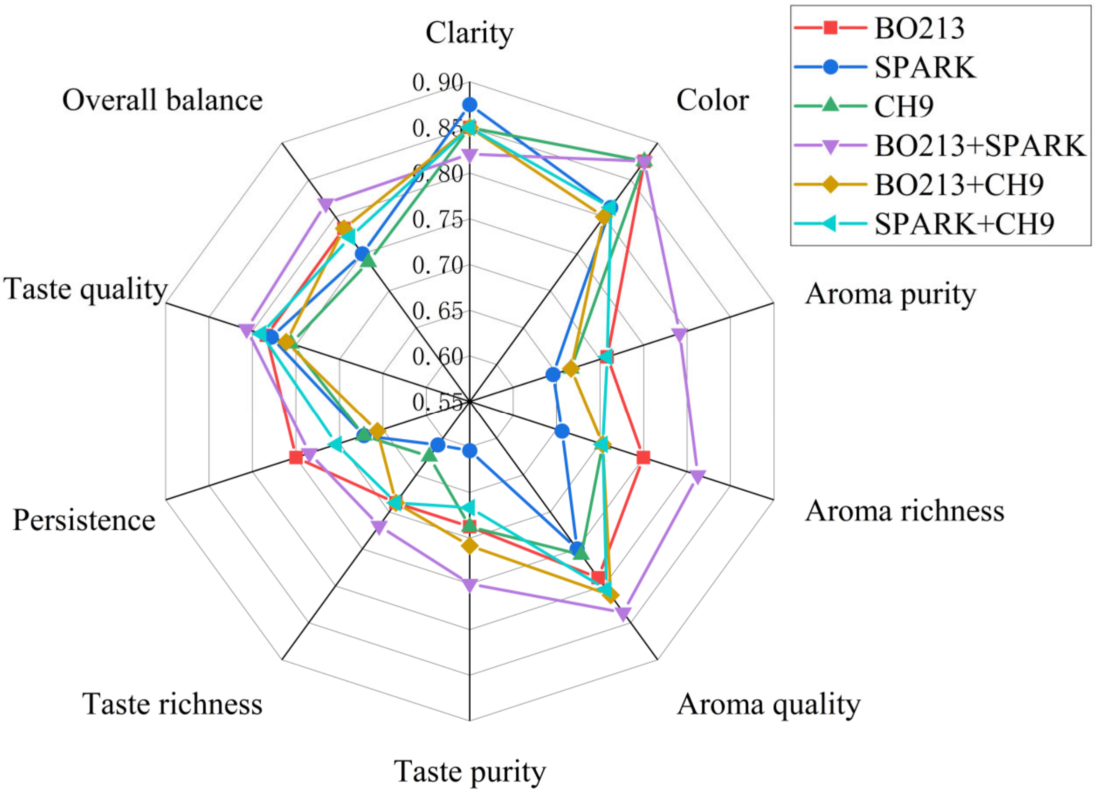

Supplementary Figure S1 Sensory analysis of persimmon wines fermented with different starters.

Supplementary Table S1 Volatile aromas in persimmon wine.

Chemicals	Retention time (min)	Area (%)	Contents(ug/L)
Esters			
Ethyl acetate	7.947	20.41	116280.5 ± 6498.6
Isobutyl acetate	11.506	0.13	79.3±5.5
Ethyl butyrate	12.292	0.02	58.7±1.8

Isoamyl acetate	15.323	2.93	319.0±24.2
Methyl caproate	17.798	0.03	1.2±0.1
Ethyl hexanoate	19.63	0.84	26.5±0.6
Ethyl heptanoate	23.409	0.02	0.8±0.1
Ethyl lactate	23.726	0.04	5025.2±162.2
Methyl caprylate	25.469	0.1	3.3±0.2
Ethyl caprylate	27.071	2.35	98.8±2.5
Methyl caprate	32.511	0.03	1.0±0.1
Ethyl caprate	33.855	0.91	41.3±0.3
Ethyl 4- decenoate	35.37	0.03	0.9±0.0
Ethyl phenylacetate	38.768	0.37	8.8±1.4
Ethyl dodecanoate	39.221	0.08	3.1±0.4
Phenyl propionate	40.368	0.05	2.0±0.4
Isopropyl myristate	43.188	0.02	0.2±0.2
Ethyl tetradecanoate	43.484	0.02	0.6±0.1
Alcohols			

2- methyl -1- propanol	14.588	0.69	4139.7±419.6
3- methyl -1- butanol	18.769	11.58	38659.8±2996.0
1-Pentanol	20.302	0.03	74.1±12.1
3-Methylpentanol	22.86	0.02	0.4±0.0
5-Methyl-2-hexanol	23.166	0.01	0.5±0.0
1-Hexanol	24.094	0.05	1.7±0.0
2-Ethylhexyl alcohol	28.89	0.05	1.6±0.0
3-Ethyl-2-pentanol	30.552	1.08	42.5±8.9
1-Octanol	31.176	0.29	4.7±0.1
6-Methyl-1-octanol	31.453	0.02	0.8±0.2
2,3-Butanediol	31.693	0.13	33333.8±959.0
5-Methyl-1-hexanol	32.917	0.04	1.3±0.0
1-Nonanol	33.855	0.91	1.9±0.1
1-Decanol	37.192	0.41	14.4±0.8
2-Phenethyl alcohol	40.879	2.65	94.2±7.7
1-Dodecanol	41.778	2.27	79.4±4.5

1-Tetradecanol	45.977	0.02	0.8±0.1
Aldehydes			
Nonanaldehyde	25.647	0.15	5.5±0.7
Dodecanal	36.005	0.06	2±0.0
2,4-Dimethylbenzaldehyde	38.902	0.09	3.3±0.3
Alkane			
1,3,5-Trioxane	36.242	0.05	1.6±0.2
Alkenes			
Phenethylene	20.564	0.07	2.7±0.3
Acids			
Acetic acid	27.37	2.38	84.1±6.0
Octanoic acid	43.846	1.18	189.1±6.2
Nonanoic acid	46.403	0.25	9.1±1.1
Ketones			
3- hydroxy -2- butanone	21.744	0.03	1.0±0.1
Nonyl methyl ketone	32.667	0.03	1.2±0.1

2-Nonadecanone	35.826	0.02	0.6±0.0
Methyl vinyl ketone	41.64	0.01	0.5±0.0
Phenols			
2-Methoxy-4-vinylphenol	46.84	0.03	1.0±0.1
2,4-Di-tert-butylphenol	49.137	0.3	11.0±1.3

The GC–MS analysis was performed as described by Li et al [1]. GC-MS-QP2020 (Shimadzu Corporation, Shanghai) was coupled in series with an olfactory detector OPV 275 (Shimadzu Corporation, Shanghai) and a DB-WAX capillary column (60 m × 0.25 mm × 0.25 μm; Agilent J&W, Santa Clara, CA, USA). The temperature of the GC column was kept as follows: 40℃ for 3 min, increase to 160℃ at a rate of 4 ℃/min, followed by an increase to 220℃ at a rate of 7℃/min, and then kept for 10 min. Electron ionization mass spectrometric data were acquired within the mass range 35–350 m/z at 0.2 s intervals combined with the selected ion monitoring mode for quantitative analysis. Aroma compounds were identified by comparing retention times, retention indexes, aroma characteristics, and mass spectra with those of standards available in the NIST 17.0 mass spectral library. The concentration of aroma compounds was quantitated by interpolating the relative area of the sample versus the area of the internal standard using calibration curves previously established for pure standards.

Compounds of chromatographical purity from Sigma-Aldrich (Shanghai, China), including aroma compounds such as ethyl acetate, isobutyl acetate, isoamyl acetate, hexyl acetate, ethyl butyrate, ethyl octanoate, n-pentyl acetate, and 2-octanol, were used as an external standard for identification and quantitation of aroma compounds in persimmon wine.

1. Li, N.; Wang, L.; Yin, J.; Ma, N.; Tao, Y. Adjustment of impact odorants in Hutai-8 rose wine by co-fermentation of *Pichia fermentans* and *Saccharomyces cerevisiae. Food Res. Int.* 2022, 153: 110959.
